# Islet cell dedifferentiation is a pathologic mechanism of long-standing progression of type 2 diabetes

**DOI:** 10.1172/jci.insight.143791

**Published:** 2021-01-11

**Authors:** Kikuko Amo-Shiinoki, Katsuya Tanabe, Yoshinobu Hoshii, Hiroto Matsui, Risa Harano, Tatsuya Fukuda, Takato Takeuchi, Ryotaro Bouchi, Tokiyo Takagi, Masayuki Hatanaka, Komei Takeda, Shigeru Okuya, Wataru Nishimura, Atsushi Kudo, Shinji Tanaka, Minoru Tanabe, Takumi Akashi, Tetsuya Yamada, Yoshihiro Ogawa, Eiji Ikeda, Hiroaki Nagano, Yukio Tanizawa

**Affiliations:** 1 Division of Endocrinology, Metabolism, Hematological Sciences and Therapeutics, Department of Medicine, Yamaguchi University Graduate School of Medicine, Ube, Yamaguchi, Japan.; 2 Department of Diabetes Research, Yamaguchi University School of Medicine, Ube, Yamaguchi, Japan.; 3 Department of Diagnostic Pathology, Yamaguchi University Hospital, Ube, Yamaguchi, Japan.; 4 Department of Gastroenterological, Breast and Endocrine Surgery, Yamaguchi University Graduate School of Medicine, Ube, Yamaguchi, Japan.; 5 Department of Molecular Endocrinology and Metabolism, Graduate School of Medical and Dental Sciences, Tokyo Medical and Dental University, Tokyo, Japan.; 6 Department of Diabetes, Endocrinology and Metabolism, National Center for Global Health and Medicine, Tokyo, Japan.; 7 Health Administration Center, Yamaguchi University Organization for University Education, Yamaguchi, Japan.; 8 Department of Molecular Biology, International University of Health and Welfare School of Medicine, Chiba, Japan.; 9 Department of Hepatobiliary and Pancreatic Surgery,; 10 Department of Molecular Oncology,; 11 Department of Diagnostic Pathology, and; 12 Department of Molecular and Cellular Metabolism, Graduate School of Medical and Dental Sciences, Tokyo Medical and Dental University, Tokyo, Japan.; 13 Department of Medicine and Bioregulatory Science, Graduate School of Medical Sciences, Kyushu University, Fukuoka, Japan.; 14 AMED-CREST, Tokyo, Japan.; 15 Department of Pathology, Yamaguchi University Graduate School of Medicine, Ube, Yamaguchi, Japan.

**Keywords:** Endocrinology, Metabolism, Beta cells, Diabetes, Insulin

## Abstract

Dedifferentiation has been implicated in β cell dysfunction and loss in rodent diabetes. However, the pathophysiological significance in humans remains unclear. To elucidate this, we analyzed surgically resected pancreatic tissues of 26 Japanese subjects with diabetes and 11 nondiabetic subjects, who had been overweight during adulthood but had no family history of diabetes. The diabetic subjects were subclassified into 3 disease stage categories, early, advanced, and intermediate. Despite no numerical changes in endocrine cells immunoreactive for chromogranin A (ChgA), diabetic islets showed profound β cell loss, with an increase in α cells without an increase in insulin and glucagon double-positive cells. The proportion of dedifferentiated cells that retain ChgA immunoreactivity without 4 major islet hormones was strikingly increased in diabetic islets and rose substantially during disease progression. The increased dedifferentiated cell ratio was inversely correlated with declining C-peptide index. Moreover, a subset of islet cells converted into exocrine-like cells during disease progression. These results indicate that islet remodeling with dedifferentiation is the underlying cause of β cell failure during the course of diabetes progression in humans.

## Abstract 2

Islet remodeling with dedifferentiation is a pathologic mechanism of β celldysfunction and lost during the course of progression in human type 2 diabetes.

## Graphical abstract

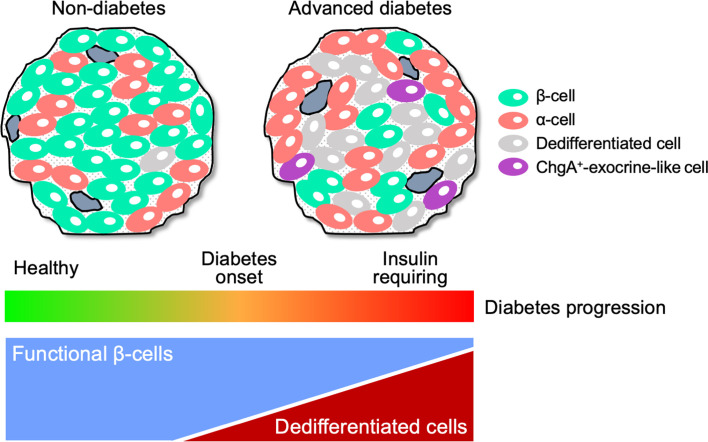

## Introduction

Diabetes mellitus (DM) is a metabolic disorder characterized by loss and dysfunction of pancreatic β cells along with insulin resistance, resulting in multiple long-term complications (1, 2). Despite various oral therapies to lower glucose, disease progression is associated with a profound decline in β cell function leading to consequent insulin replacement (3, 4). Although it is accepted that increased apoptosis plays a role in declining β cell function and mass over time (5–8), an increase in apoptosis alone is insufficient to explain the β cell deficit in type 2 diabetes (9, 10). Additionally, in human islet-to-mouse studies, neither chronic hyperglycemia nor peripheral insulin resistance was sufficient to cause apoptosis in human islets (11). Definitive lineage-tracing experiments in rodent diabetes have recently identified β cell dedifferentiation with reversion to a progenitor-like state (12, 13). Importantly, analyses of human cadaveric pancreatic tissues have provided corroboration of the results of animal studies (14, 15). A dedifferentiated β cell is defined as one that no longer contains pancreatic hormones, although it retains endocrine features such as chromogranin A and/or synaptophysin immunoreactivity (12, 14). The fact that islet hormone-negative endocrine cells were significantly increased in postmortem diabetic specimens suggests β cell dedifferentiation to be involved in insufficient insulin secretion (14). However, the information from cadaveric sample analyses does not account for either the extent or the mechanism by which β cell dedifferentiation contributes to the β cell deficit and its progression during the entire disease course of diabetes. Elucidating this issue is a key challenge owing to the difficulty in obtaining long-term access to human pancreatic samples of appropriate quality for analyses, in conjunction with the necessary medical history and metabolic profiles. Therefore, we took advantage of the opportunity to examine pancreatic samples from Japanese patients with and without diabetes undergoing partial pancreatectomy, for resection of pancreaticobiliary neoplasms, to examine the hypothesis that islet remodeling with dedifferentiation is a pathologic mechanism underlying long-standing disease progression. To this end, we subclassified the subjects with diabetes into early- and advanced-stage disease and examined islet morphology and dedifferentiation, as well as correlations with clinical parameters. Our work reveals the involvement of dedifferentiation in β cell dysfunction and loss during the course of diabetes progression.

## Results

### Clinical data and morphometric measurements.

Clinical characteristics of the 11 control patients (non-DM) and 26 diabetic patients subclassified into 3 categories are summarized in Table 1, and clinical feature of each individual subject is shown in Supplemental Table 1 (supplemental material available online with this article; https://doi.org/10.1172/jci.insight.143791DS1). All study groups included patients with pancreatic cancer, and there were several with cholangiocarcinoma and duodenal papilla cancer among the non-DM and early- and advanced-DM patients. The non-DM and intermediate-DM patients were younger than those in the other 2 groups, although the mean ages did not differ significantly, whereas mean ages were similar in the early- and advanced-DM patients. Mean maximum BMI differed minimally among groups. However, BMI decreased with disease progression and was significantly lower in the advanced-DM than in the non-DM subjects (*P* = 0.031). Fasting plasma glucose and glycated hemoglobin A1c (HbA1c) levels in the non-DM patients showed normal glucose tolerance. Durations of diabetes in the early-, advanced-, and intermediate-DM subjects were 4.2 ± 0.9, 17.5 ± 5.6, and 8.0 ± 1.4 years, respectively. The preoperative HbA1c levels were 6.7% ± 0.4 % (50 ± 5.5 mmol/mol), 7.3% ± 0.7% (57 ± 7.9 mmol/mol), and 7.3% ± 0.9% (56 ± 10.1 mmol/mol), respectively. There were no statistically significant differences in HbA1c levels among the diabetic groups. As diabetes progressed, fasting plasma glucose levels rose and C-peptide immunoreactivity (CPR) levels decreased, resulting in a profound C-peptide index (CPI) reduction in patients with advanced DM (*P* = 0.0005 vs. non-DM).

Subjects with diabetes had a broad range of fractional β cell, α cell, and islet areas and α cell/β cell ratios as compared with non-DM subjects, but the differences among all groups did not reach statistical significance (Supplemental Figure 1, A–D). Although not statistically significant, islet areas in subjects with diabetes tended to be increased in the body to tail as compared with the head of the pancreas (*P* = 0.057) (Supplemental Figure 1E). There were significant correlations among β cell, α cell, and islet areas (*r* = 0.818, *P* = 3.431 × 10^–9^; *r* = 0.863, *P* = 5.375 × 10^–11^, respectively) (Supplemental Figure 1, F and G). There was also a significant correlation between the β cell and α cell areas (*r* = 0.717, *P* = 1.802 × 10^–6^) (Supplemental Figure 1H). In addition, there was a weak but statistically significant correlation between the α cell/β cell ratio and islet area (*r* = 0.378, *P* = 0.028) (Supplemental Figure 1I).

### Diabetic islets have preserved endocrine cells but show altered β cell and α cell fractions.

**Figure 1:**
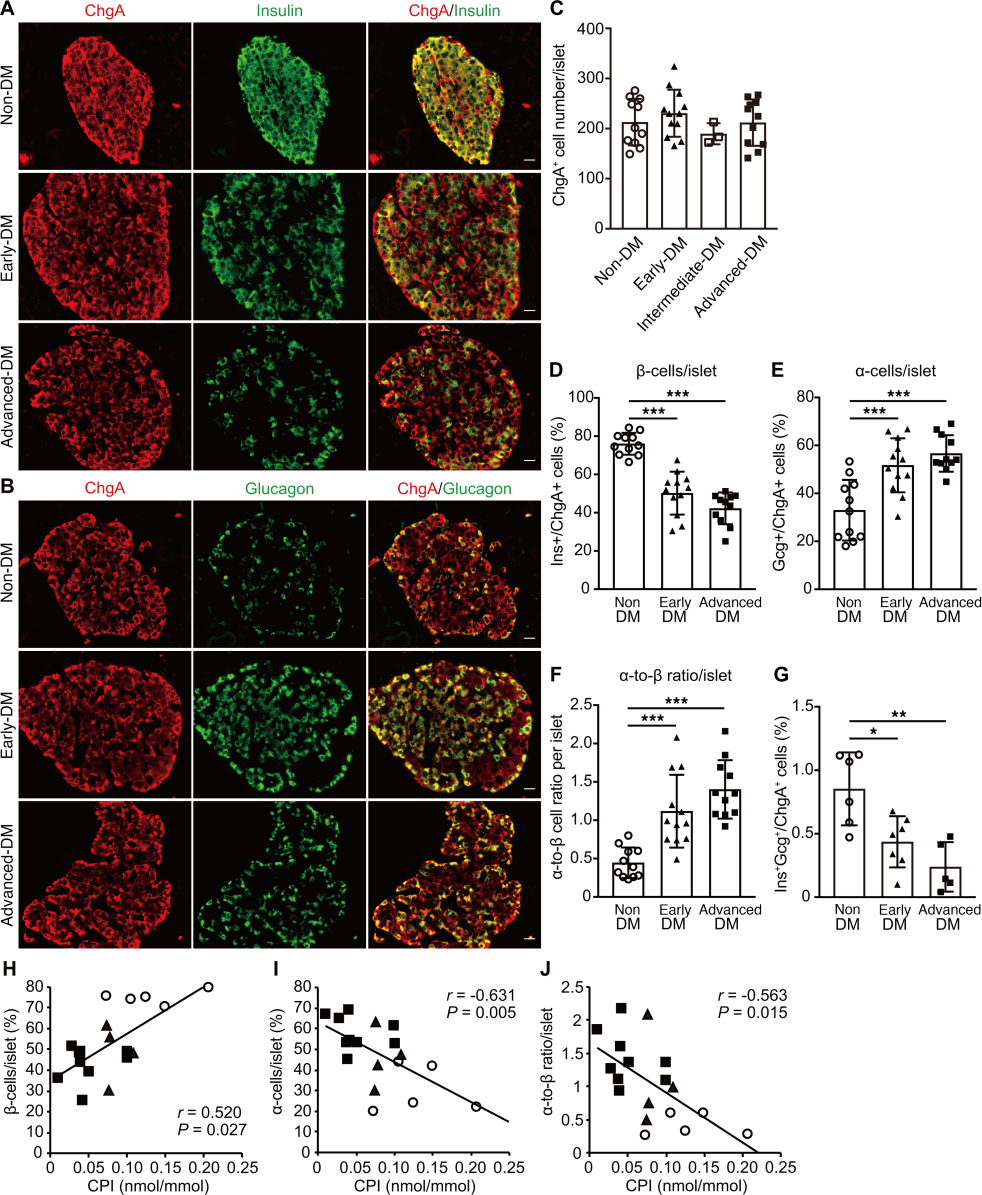
Morphologic changes in individual islets and correlation with β cell function. Representative images of pancreatic islets immunostained with chromogranin A (ChgA) (shown in red) and (**A**) insulin (shown in green) and (**B**) glucagon (shown in green) in each group. Scale bar: 20 μm. Quantitative analyses of (**C**) ChgA-positive cells per islet, (**D**) β cells per islet, (**E**) α cells per islet, and (**F**) the α cell/β cell ratio per islet. Data are means ± SD (*n* = 11 for non-DM, *n* = 12 for early-DM, *n* = 11 for advanced-DM, and *n* = 3 for intermediate-DM). ***P *<* 0.001 by 1-way ANOVA followed by Bonferroni’s post hoc test. (**G**) Quantitative analyses of insulin and glucagon double-positive cells in the pancreatic sections of specimens from the indicated subjects in each group. Data are means ± SD (*n* = 6 for non-DM, *n* = 7 for early-DM, and *n* = 5 for advanced-DM). *P *<* 0.05, **P *<* 0.01 by 1-way ANOVA followed by Bonferroni’s post hoc test. We performed single regression analysis (Spearman’s correlation coefficient) to assess relationships between the C-peptide index obtained from 18 subjects (*n* = 5 for non-DM, *n* = 4 for early-DM, and *n* = 9 for advanced-DM) and (**H**) β cells/islet, (**I**) α cells/islet, and (**J**) α cell/β cell ratio per islet. Open circles, non-DM control subjects. Closed triangles, early-DM subjects. Closed squares, advanced-DM subjects. CPI, C-peptide index.

The large deviations in islet morphometrics in subjects with diabetes may reflect diverse capabilities for islet compensation in response to metabolic demands. To gain pathologic insight into failing islets, we examined the morphology of individual islets. Representative images demonstrate changes in the appearance of size-matched islets stained with chromogranin A (ChgA) and insulin or Gcg, with disease progression (Figure 1, A and B). ChgA-positive cells per islet numbers were similar in all groups (Figure 1C). Because of the small number of subjects, we did not include the intermediate-DM group in further comparisons. We detected a 34% decrease (from 76% to 50%) and a 44% decrease (from 76% to 42%) in β cells/islet ratio in the early-DM and advanced-DM groups, respectively, as compared with non-DM subjects (*P* *<* 0.0001, *P* *<* 0.0001) (Figure 1D). The α cells/islet ratio increased by 58% (from 33% to 52%) and 73% (from 33% to 57%) in the early-DM and advanced-DM groups, respectively (*P* = 0.007, *P* *<* 0.0001), leading to a higher α cell/β cell ratio per islet (Figure 1, E and F). Contrary to a previous report (16), the ratio of insulin/Gcg double-positive cells, when normalized by the number of ChgA-positive cells, was significantly decreased in diabetic groups as compared with non-DM subjects (Figure 1G). In addition, the mean ratio of advanced-DM was further decreased, by 55%, as compared with that of early-DM, suggesting that cells coexpressing immunoreactive insulin and glucagon were hardly detectable in islets of diabetic subjects. Next, we sought to determine whether there are any functional correlations with alterations in islet morphology. We found a significant correlation between a decrease in β cell/islet ratio and decreased CPI (*r* = 0.520, *P* = 0.027) (Figure 1H). In contrast, the α cell/islet ratio and the α cell/β cell ratio per islet both showed inverse correlations with CPI (*r* = –0.631, *P* = 0.005; *r* = –0.563, *P* = 0.015, respectively) (Figure 1, I and J).

### Islet cell dedifferentiation is involved in diabetes progression.

**Figure 2:**
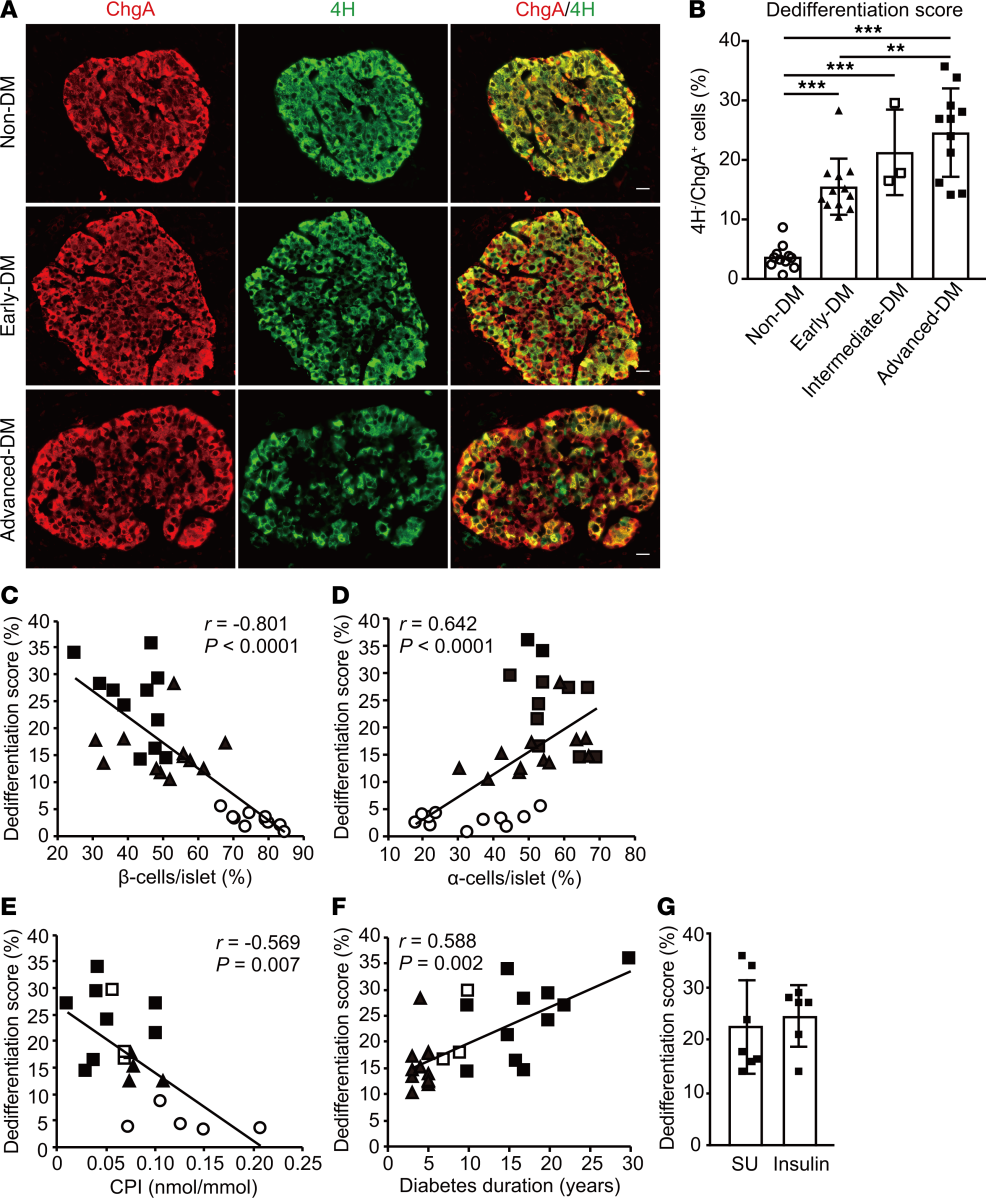
Evaluation of dedifferentiation and correlations with islet morphology and clinical parameters. (**A**) Representative images of pancreatic islets immunostained with ChgA (shown in red) and endocrine cocktail (insulin, glucagon, somatostatin, and pancreatic polypeptide [4H], shown in green). Scale bar: 20 μm. (**B**) Quantitative analysis of dedifferentiated cells (dedifferentiation score), calculated as ChgA^+^4H^–^/ChgA^+^ cells per islet. Data are means ± SD. **P *<* 0.01, ***P *<* 0.001 by 1-way ANOVA followed by Bonferroni’s post hoc test (*n* = 11 for non-DM, *n* = 12 for early-DM, *n* = 3 for intermediate-DM, and *n* = 11 for advanced-DM). Single regression analysis (Spearman’s correlation coefficient) of correlations between dedifferentiation score and (**C**) β cells/islet, (**D**) α cells/islet, and (**E**) the C-peptide index obtained from 21 subjects (*n* = 5 for non-DM, *n* = 4 for early-DM, *n* = 3 for intermediate-DM, and *n* = 9 for advanced-DM), and (**F**) diabetes duration. (**G**) Comparison of dedifferentiation score between the subjects receiving insulin treatment (*n* = 6) and those treated with sulfonylurea but not insulin (*n* = 7). Open circles, control subjects. Closed triangles, early-DM subjects. Open squares, intermediate-DM subjects. Closed squares, advanced-DM subjects. CPI, C-peptide index. SU, sulfonylurea.

We next sought to establish a link between dedifferentiation and loss and/or dysfunction of β cells during the course of the disease. We arbitrarily defined a dedifferentiated cell as a ChgA-positive cell that failed to react with the antibodies against the aforementioned 4H (Figure 2A). Ghrelin, another pancreatic hormone, was separately tested. The percentages of cells immunoreactive for ghrelin in ChgA-positive cells was less than 1% without a significant change among study groups (data not shown). We next determined the numbers of dedifferentiated cells per islet in all study subjects. As shown in Figure 1C, there was no loss of cells corresponding to the general endocrine features in type 2 diabetes. The percentages of cells positive for ChgA and negative for 4H rose progressively, being lowest in the non-DM, then rising from early-DM, through intermediate-DM, and finally being highest in the advanced-DM specimens (4% ± 2% vs. 16% ± 5%, 21% ± 7%, 25% ± 7%, respectively) (*P* *<* 0.001) (Figure 2B). Percentages of dedifferentiated cells (dedifferentiation score) in intermediate-DM and advanced-DM were higher by 37% and 59% versus the early-DM group, respectively (*P* = 0.618, *P* = 0.0016, respectively). There was no significant difference in dedifferentiation scores between the head and body to the tail of pancreas (Supplemental Figure 2A). We also assessed insulin-positive cells immunoreactive for nuclear V-maf musculoaponeurotic fibrosarcoma oncogene homolog A (MAFA) and NK homeobox, family 6, member A (NKX6.1), in a subset of subjects and found similar declines in early- and advanced-DM (Supplemental Figure 2, B, C, E, and F). When normalized by the number of ChgA-positive cells, although no significant differences were seen among the diabetic groups, there was a tendency for nuclear MAFA and NKX6.1, immunoreactivity to decrease with disease progression (Supplemental Figure 2, D and G). Notably, immunoreactivities for nuclear MAFA and NKX6.1 were weaker in advanced- than in early-DM specimens, demonstrating a loss of a mature cellular identity in parallel with dedifferentiation. In all study groups, dedifferentiation scores correlated strongly with a decreased β cell/islet ratio (*r* = –0.801, *P* = 1.297 × 10^–8^) and an increased α cell/islet ratio (*r* = 0.642, *P* = 4.333 × 10^–5^) (Figure 2, C and D). The morphologic correlations were further confirmed by immunostaining with insulin and other pancreatic hormones separately (Supplemental Figure 3A). Functional relevance was next examined. Dedifferentiation scores inversely correlated with CPI in all study groups (*r* = –0.569, *P* = 0.007) (Figure 2E), and a similar inverse correlation was shown in the diabetic subjects except those with advanced-DM (*r* = –0.7928, *P* = 0.0397) (Supplemental Table 2). As to clinical relevance, whereas no significant correlation between dedifferentiation scores and disease duration was identified for each diabetic group (Supplemental Table 2), dedifferentiation scores correlated significantly with disease duration when tested in whole subjects with diabetes (Figure 2F). In 13 subjects, there was no difference in dedifferentiation score between treating with insulin and with sulfonylurea (Figure 2G). In addition, there was a significant correlation between BMI prior to operation and dedifferentiation score in nondiabetic control subjects (*r* = –0.676, *P* = 0.026), whereas it was not seen in those with diabetes (Supplemental Figure 3, B–D).

### Correlations of dedifferentiation with islet morphology and age in early-DM subjects.

**Figure 3:**
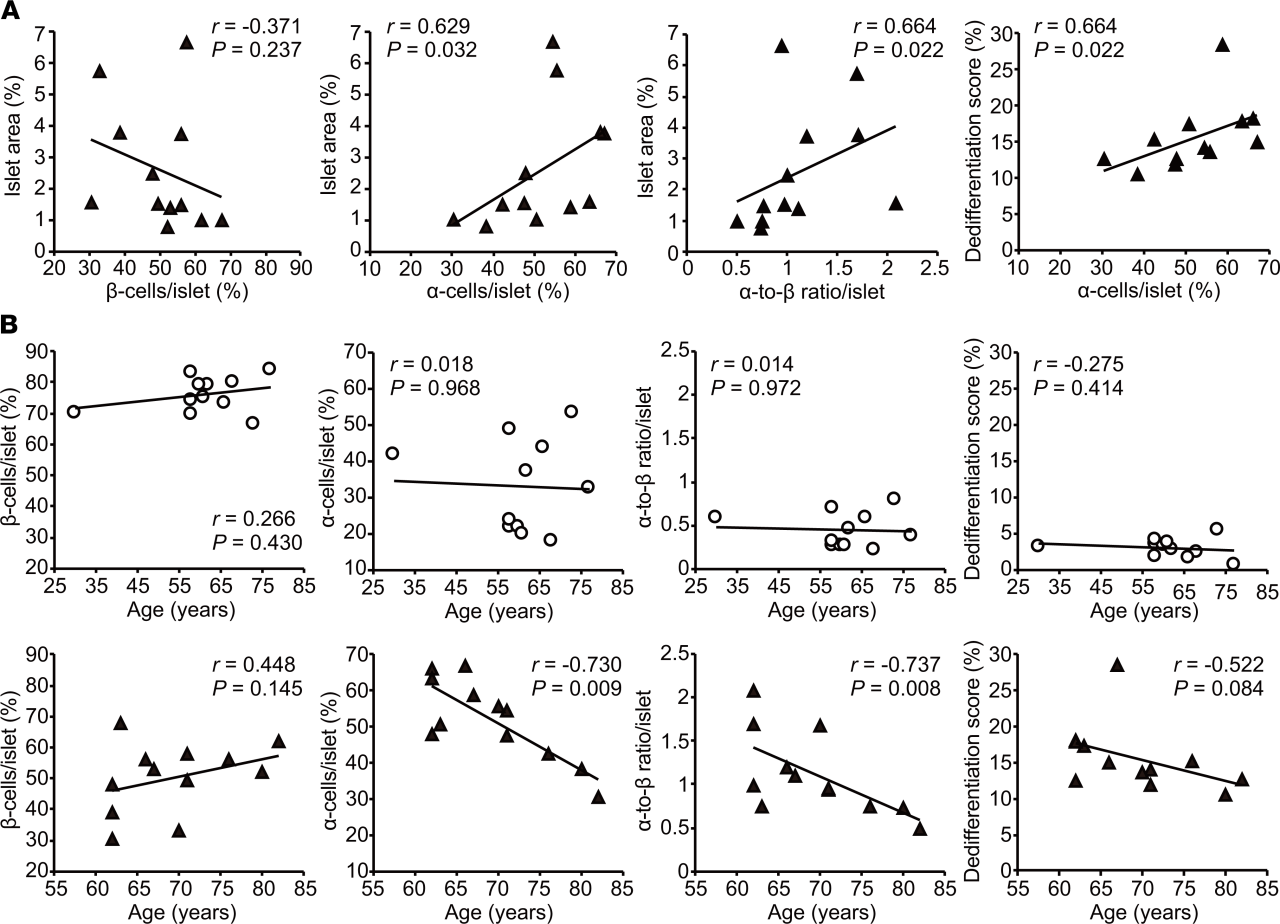
Analysis of correlations of dedifferentiation with islet morphology and age in early-DM subjects. (**A**) Single regression analysis (Spearman’s correlation coefficient) of correlations between islet morphology parameters and islet area with dedifferentiation scores in early-DM subjects (*n* = 12). (**B**) Single regression analysis (Spearman’s correlation coefficient) of correlations between age and islet plasticity parameters in non-DM control (*n* = 11) and early-DM subjects (*n* = 12). Open circles, non-DM control subjects. Closed triangles, early-DM subjects.

β cell dysfunction with a background of insulin resistance causes hyperglycemia that would in turn promote β cell dedifferentiation (12–14, 17–19). However, other factors that might be involved are not fully understood. To explore this issue, we focused on 12 early-DM subjects, who would presumably have fewer long-standing effects of hyperglycemia than patients with longer disease durations. As shown in Figure 3A, we found that an increased islet area correlated significantly with α cell/islet ratio rather than β cell/islet ratio in early-DM, resulting in an increase in the α cell/β cell ratio per islet. A significant correlation between dedifferentiation score and α cell/islet ratio was demonstrated (*r* = 0.644, *P* = 0.022). However, no increase in the frequency of interconversion between β cells and α cells, partly represented by insulin/Gcg double-positive cells, was identified in diabetic patients (Figure 1G), raising the possibility of an indirect correlation. Therefore, this series of observations implied that islet area expansion, assumed to occur before progression of hyperglycemia, would be accompanied by islet remodeling owing to islet cell plasticity. This would result in α cells being increased and thereby correlating indirectly with dedifferentiation. We next tested correlations with age (Figure 3B). All tests on control subjects showed there were no correlations between islet morphometries and age. Although there was no significant correlation between β cells/islet and age, we found α cell/islet ratio and α cell/β cell ratio per islet to correlate inversely with age. Moreover, dedifferentiation scores were decreased in older subjects and appeared to be inversely associated with age, although the relationship did not reach statistical significance (*r* = –0.522, *P* = 0.084).

### Advanced stage of diabetes and altered islet cell fate.

**Figure 4:**
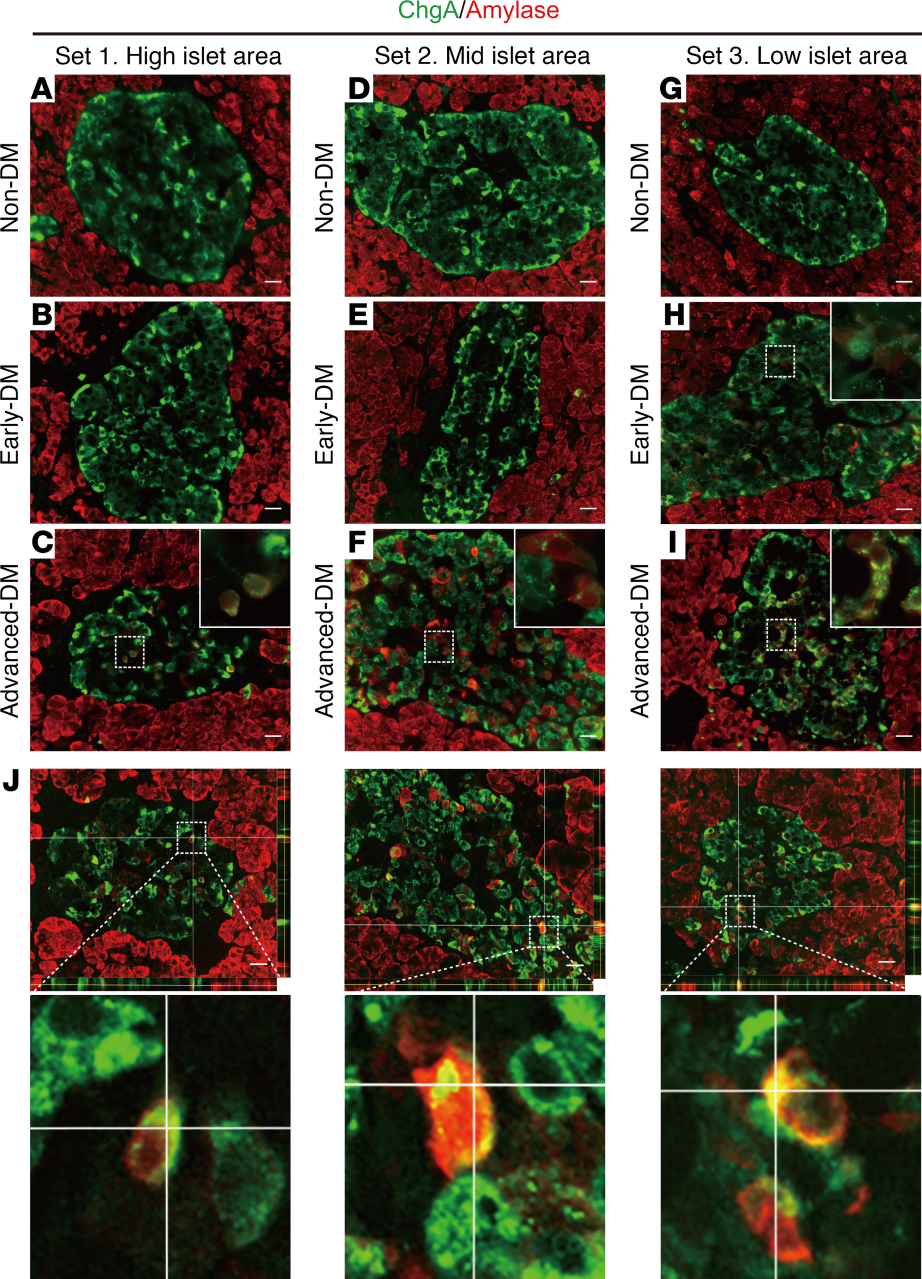
Immunohistochemical evidence of conversion from endocrine to exocrine cell phenotype in failing islets. Islet area–matched male subjects without pancreatic cancer were selected from each group. The 9 subjects were classified into 3 comparison sets according to the fraction of islet area as shown in Supplemental Table 3. Their pancreatic sections were examined. Representative images of pancreatic islets immunostained with ChgA (green) and amylase (red) of 9 subjects in 3 comparison sets representing different islet areas are shown (**A**–**C**, **D**–**F**, and **G**–**I**). Insets demonstrate representative cells showing immunoreactivity for ChgA and amylase. Scale bar: 20 μm. (**J**) *Z*-stack of pancreatic islets immunostained with ChgA (green) and amylase (red) of advanced-DM. Multiple *Z*-plane fluorescent images of pancreatic sections of the subjects in **C**, **F**, and **I** were captured. The representative *Z*-stack images are shown in the same order as in **C**, **F**, and **I**. The representative islet cells expressing both amylase (red) and ChgA (green) are shown in the lower images with a high magnification. Scale bar: 20 μm.

We then sought to examine features of dedifferentiated cells in the advanced disease stage. Tissues of islet area–matched male subjects without pancreatic cancer, whose details are summarized in Supplemental Table 3, were costained with ChgA and a nonendocrine marker, i.e., amylase, and the results were then compared among all groups. In 3 sets classified with different islet areas (Figure 4, A–C, D–F, and G–I, respectively), all advanced-DM subjects exhibited a subset of islet ChgA-positive cells immunoreactive for amylase (Figure 4, C, F, and I). *Z*-stack analysis demonstrated that amylase and ChgA were expressed in virtually the same cells (Figure 4J). An early-DM subject, who had the highest dedifferentiation score in this group, had similar cellular findings (Figure 4H). Notably, such cells showed apparently decreased immunoreactivities for ChgA as compared with neighboring amylase-negative cells in the same islet, suggesting that a subset of dedifferentiated cells loses endocrine features and takes an exocrine fate. Although a previous study demonstrated a mesenchymal marker, vimentin-expressing cells emerging in diabetic donor islets (20), no islet cells immunoreactive for vimentin were detectable in our cases (data not shown). In contrast to early-DM (Figure 3A), the α cells/islet ratio in advanced-DM tended to show an inverse association with dedifferentiation score (Supplemental Figure 4A). Immunohistochemical analysis using a specific anti–glucagon-like peptide-1 (GLP-1) antibody, which is highly selective for processed amidated GLP-1 directed to the COOH-terminal (21), demonstrated that glucagon colocalizes with GLP-1 and that glucagon immunoreactivities relative to GLP-1 decreased with disease progression (Supplemental Figure 4B). This immunohistochemical evidence suggested that both β cell and α cell identity may be impaired over the course of disease progression.

## Discussion

The pathogenesis of β cell failure integrates both functional and quantitative (cell number) defects. Islet exerts plasticity in diverse metabolic conditions (18, 19, 22), and it has been suggested that β cells undergo dedifferentiation in type 2 diabetes (14, 16, 17, 23). In light of the human evidence, we sought to elucidate the relevance of dedifferentiation to disease progression during the course of diabetes. Our study using surgically resected pancreatic specimens, taken into consideration with clinical information, demonstrated that islet plasticity in diverse disease conditions, which leads to dedifferentiation, is a pathologic basis of β cell failure over the entire course of type 2 diabetes.

From a clinical standpoint, our study had the following potentially novel findings. First, the clinical relevance of dedifferentiation to long-standing progression of diabetes was demonstrated. Second, islet cells undergo dedifferentiation in the early stage of diabetes, in association with β cell dysfunction. Finally, a subset of dedifferentiated cells exhibited an exocrine-like phenotype, in association with profound dysfunction. Despite the large deviations of percentage areas of β cell, α cell, and islet relative to whole pancreas area, loss of β cells and expansion of α cells in individual islets and their correlation with dedifferentiation are the common features of islets of patients with diabetes. Importantly, a link between dedifferentiation and dysfunction was demonstrated in association with disease progression. Despite loss of β cells, numbers of islet endocrine cells, as assessed based on ChgA immunoreactivity, were preserved even in subjects with advanced DM, implying an increase in β cells that had lost mature identity and the capacity for insulin production. This was supported by transcription factor analysis demonstrating progressive loss of nuclear MAFA and NKX6.1 as insulin-positive cells decreased. In contrast to previous studies (14, 16), cells with mixed α and β features, which were partly represented by insulin/Gcg double positivity, were undetectable in our study. This may suggest that β cells are unlikely be a source of α cells, at least in Japanese subjects with diabetes. In fact, a recent study reported a similar observation in surgically resected pancreas from Japanese subjects with long-standing diabetes (24).

In rodents, dedifferentiated β cells revert to a progenitor-like stage characterized by transcription factor expression (12, 13, 25). As previously described (14), we were also unable to detect neurogenin3 immunoreactivity in islets. Aldehyde dehydrogenase 1 isoform A3 (ALDH1A3) has been identified as an alternative progenitor marker in failing islets (14, 26). However, given the nature of ALDH1 in cancer progenitor cells (27), we did not assess the significance of ALDH1A3 to avoid implications that would lack the certainty necessary for this analysis. In fact, pancreatic cancer tissues from our cases showed strong immunoreactivities for ALDH1A3 (data not shown). Thereby, understanding to what extent islet cells become dedifferentiated was limited. Moreover, we observed that a subset of islet endocrine cells exhibited an exocrine-like phenotype, in association with disease progression. This finding may provide pathologic insight into the irreversible and profound dysfunction in advanced disease. Further work exploring the association between the stage of dedifferentiation and functionality is needed to gain key mechanistic insight into failing islets.

When is dedifferentiation initiated? We observed that β cells undergo dedifferentiation, starting early in the disease course. In this regard, a correlation between dedifferentiation and increased α cells was observed in early-DM subjects, and the increasing α cell/islet ratio paralleled expansion of islet area (Figure 3A). As reported previously, nondiabetic subjects with insulin resistance showed increased numbers of α cells and β cells with a consequent increase in islet volume (22, 28), suggesting that expansion of α cells in early DM could, at least partly, result from preexisting insulin resistance. Therefore, we speculate that when faced with sustained hyperglycemia, islet cells exert plasticity favoring dedifferentiation, possibly potentiated by insulin resistance. In addition, whereas our subject number was small, we did not observe that aging promoted either dedifferentiation or expansion of α cells. This allows us to hypothesize that islet cell plasticity declines with aging, although investigations with a larger number of subjects will be needed to examine this hypothesis.

Loss of maturity through dedifferentiation provides a pathophysiologic link between dysfunction and reduced β cell numbers. Despite the evidence that reversal of hyperglycemia can partially restore β cell function even in advanced disease (29), the potential for functional recovery declines with disease progression, ultimately leading to irreversible insulin insufficiency (18). Our observation that dedifferentiation scores did not differ between treatments with insulin and sulfonylurea in advanced disease suggest that even decreasing the β cell overload by insulin therapy would not reverse dedifferentiation, once diabetes has reached an advanced disease stage. Therefore, intervention in the early disease stage against insulin resistance as well as hyperglycemia reduces metabolic overloading of β cells and thereby offers an opportunity for recovery, thus preventing a devastating and irreversible loss of β cells (18, 30–32). Nonetheless, for β cells themselves, adopting dedifferentiation is advantageous. This seemingly “selfish” behavior facilitates the survival of β cells that are stressed continuously by metabolic overloads during the long-term course of diabetes.

Our study has limitations. Subjects with various pancreaticobiliary tumors were selected by applying stringent exclusion criteria to reduce potential tumor-related effects on disease conditions and/or progression, and we carefully sectioned the tissues with avoidance of nearby tumors. However, pancreatic cancer in particular may have affected islet plasticity and morphology through indirect mechanisms, which would be a limitation of this study (33).

In conclusion, our observations highlight islet cell dedifferentiation as a mechanism underlying diabetes progression. This makes dedifferentiation a potential target for treatments. Future studies exploring mechanistic insight into islet plasticity with dedifferentiation are warranted and may open new avenues to the prevention and/or reversal of β cell failure in type 2 diabetes.

## Methods

### Research design.

Archived pancreatic samples from 26 Japanese subjects with type 2 diabetes (23 males and 3 females) and 11 nondiabetic control subjects (9 males and 2 females), who had undergone partial pancreatectomy (19 PD, 18 DP) were examined for resection of pancreaticobiliary tumors. A total of 29 patients had been operated on at the Department of Gastroenterological Surgery, Yamaguchi University Hospital, Ube, Japan, 8 at the Department of Hepatobiliary and Pancreatic Surgery, Tokyo Medical and Dental University Hospital, Tokyo, Japan. We retrospectively selected subjects according to their medical histories and records.

### Description of subjects.

All subjects had been overweight (BMI ≥ 25 kg/m^2^) during adulthood, and none had a family history of diabetes. Patients administered anticancer drugs and glucocorticoids prior to pancreatic resection, those with functional pancreatic endocrine tumors, those with a medical history of pancreatitis and excessive alcohol consumption, and those with renal failure (estimated glomerular filtration rate < 30 mL/min/1.73 m^2^) were all excluded from this study. Based on fasting and casual blood glucose and HbA1c measured 1–14 days before pancreatic resection, glycemic status was confirmed to meet the criteria for diabetes or normal glucose tolerance defined by the Japan Diabetes Society (34). One nondiabetic patient whose glycemic status was validated by a 75 g oral glucose tolerance test lacked an HbA1c measurement. To preclude tumor-related diabetes, only patients who had been diagnosed with type 2 diabetes by a diabetologist and had been under medical management for at least 3 years were enrolled. Furthermore, patients with a relative increase in HbA1c of more than 1% (11 mmol/mol) in the 6 months prior to surgery and those with a preoperative HbA1c of more than 8.6% (70 mmol/mol) were excluded. Patients in whom either insulin or insulin secretagogues had been newly administered owing to worsening glycemic control in the 12 months before pancreatic resection were also excluded. Subjects with diabetes were subclassified into 3 disease progression categories, early-DM, advanced-DM, and intermediate-DM, according to medical records. Early-DM was defined as a disease duration of less than 5 years, no medical history of microvascular complications, and never having been treated with insulin and/or sulfonylurea. Advanced-DM was defined as a disease duration of more than 10 years, any medical history of microvascular complications, and continuous treatment with sulfonylurea or insulin for at least 1 year. Intermediate-DM was defined by exclusion from the other categories. In a subset of subjects (5 non-DM, 4 early-DM, 9 advanced-DM, and 3 intermediate-DM), preoperative serum fasting CPR and blood glucose were concomitantly measured. To assess insulin secretory reserve, we calculated the CPI using the following formula: fasting CPR/fasting plasma glucose (35). Features of individual subjects are shown in Supplemental Table 1.

### Pancreatic tissue processing and immunohistochemistry.

Pancreatic samples obtained from the edge of the surgically resected portion had been fixed, paraffin-embedded, and archived. The tissues were serially sectioned at a thickness of 4 μm at 2 random levels and processed for histological analysis. All hematoxylin and eosin–stained pancreatic sections were confirmed by in-house pathologists to contain no pancreatic tissues with tumor elements, pancreatitis and/or autolysis, and immunohistochemistry was then performed. Nuclear counterstaining of sections was performed with DAPI (Vector Laboratories). Histochemical reactions were assessed at the same time in all groups studied, using the same lot of antibodies at dilutions and light exposure times predetermined to maximize sensitivity and minimize nonspecific staining. To identify islet dedifferentiated cells, the tissues were stained with a cocktail of 4 major pancreatic hormones (4H: insulin, glucagon, somatostatin, and pancreatic polypeptide) and ChgA according to a previous report (14). All antibodies used for immunohistochemical analysis are listed in Supplemental Table 4. Fluorescence images were captured using a BZ-X710 microscope with BZ-X software (Keyence).

### Morphometric analysis.

To perform quantitative analyses, we analyzed 2 random sections per subject. We also randomly selected 20 islets with a long diameter of 150–400 μm over pancreas area per section and captured them to allow analysis of individual islets. For determination of β cell, α cell, and islet areas, the entire pancreatic section was imaged, and the total area of the pancreas was measured with removal of interlobular connective tissue, large blood vessels, and adipocytes. The immunoreactive areas for insulin, glucagon, and ChgA were then determined. β cell, α cell, and islet areas were expressed as a percentage of the total pancreatic area, and the ratio of α cell area to β cell area was represented as the α cell/β cell ratio. The numbers of insulin-, Gcg-, and ChgA-positive cells per islet were scored according to the DAPI-stained nucleus. The fractions of insulin- and Gcg-positive cells to ChgA-positive cells were represented as β cells/islet and α cells/islet, respectively. The ratio of α cells/islet to β cells/islet was represented as the α cell/β cell ratio per islet. The ChgA-positive and 4H-negative cells were scored as dedifferentiated cells, as previously reported (14). We captured islets containing at least 1 dedifferentiated cell and measured percentage of dedifferentiated cells in ChgA-positive cells per islet, representing it as dedifferentiation score. The number of insulin/Gcg double-positive cells per islet was measured in subjects less than 70 years of age whose α cell/β cell ratio per islet score was within the mean ± 0.5 SD. In subjects whose dedifferentiation scores were within the mean ± 0.5 SD, we scored insulin-positive cells stained for nuclear MAFA and NKX6.1. For quantitative analysis of insulin/Gcg double-positive cells and transcription factors in β cells, at least 2000 insulin-positive cells per subject were examined. Supplemental Table 1 shows the details of the analyzed subjects. All quantitative measurements were conducted using BZ-X software in a blinded fashion by 2 in-house independent investigators.

### Statistics.

Quantitative data are presented as means ± SD. Significant differences were evaluated by 1-way ANOVA followed by Bonferroni’s post hoc test. To assess correlations among morphometric data and with clinical parameters, single regression analysis was carried out with Spearman’s correlation coefficient. A *P* value of less than 0.05 was considered significant. All statistical analyses were performed with GraphPad Prism software version 7.

### Study approval.

The collection and use of clinical data, followed by analysis of pancreatic samples, were approved by the ethics review board of Yamaguchi University Hospital (approval number H29-112, H30-176) and Tokyo Medical and Dental University Hospital (approval number M2000-1890). All subjects provided appropriate written informed consent.

## Author contributions

KAS and K Tanabe prepared the manuscript. K Tanabe and YT designed this study. KAS, K Tanabe, HM, RH, T Takagi, TF, T Takeuchi, RB, MH, K Takeda, and SO obtained the data. KAS and K Tanabe wrote the manuscript. KAS, K Tanabe, and YT edited the manuscript. YT reviewed the manuscript. HM, AK, ST, MT, and HN performed partial pancreatectomy procedures. KAS, K Tanabe, YH, RH, T Takeuchi, WN, and EI performed pathologic analyses of pancreatic specimens. TY, YO, EI, HN, and YT contributed to interpretation of the data. All authors were involved in discussing the results and providing commentary on the manuscript. K Tanabe and YT had full access to all of the data in the study and take responsibility for data integrity and accuracy of data analysis. YT is the guarantor of this work.

## Supplementary Material

Supplemental data

## Figures and Tables

**Table 1:**
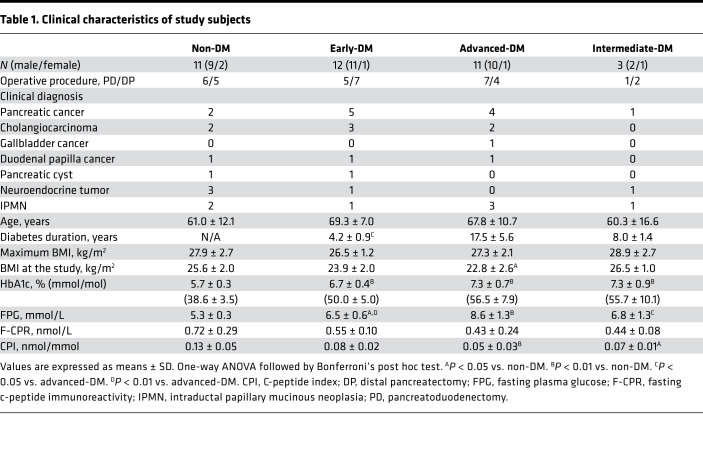
Clinical characteristics of study subjects

## Version 1 01/11/2021

Electronic publication
