# Supplementary material for: Islet cell dedifferentiation is a pathologic mechanism of long-standing progression of type 2 diabetes
Source: JCI Insight. 2021 Jan 11;6(1):e143791. doi: 10.1172/jci.insight.143791 (PMC7821596; doi:10.1172/jci.insight.143791)
Supplement: Supplemental data [file jciinsight-6-143791-s080.pdf]

Islet cell dedifferentiation is a pathologic mechanism of longstanding progression of type 2 diabetes

Kikuko Amo-Shiinoki, Katsuya Tanabe, Yoshinobu Hoshii, Hiroto Matsui, Risa Harano, Tatsuya Fukuda, Takato Takeuchi, Ryotaro Bouchi, Tokiyo Takagi, Masayuki Hatanaka, Komei Takeda, Shigeru Okuya, Wataru Nishimura, Atsushi Kudo, Shinji Tanaka, Minoru Tanabe, Takumi Akashi, Tetsuya Yamada, Yoshihiro Ogawa, Eiji Ikeda, Hiroaki Nagano and Yukio Tanizawa

Supplemental Information

Contents:

Supplemental Figure 1; Morphometric analysis of islets in pancreatic tissue sections from non-diabetic subjects and diabetic subjects

Supplemental Figure 2; The percentages of dedifferentiated cells in distinct pancreatic portions and analysis of two transcription factors, MAFA and NKX6.1

Supplemental Figure 3; Fluorescent image of islets stained separately for ChgA, insulin and pancreatic hormones and correlations between dedifferentiation score and BMI for each group

Supplemental Figure 4; Alterations in α-cells with disease progression

Supplemental Table 1; Clinical characteristics of individual study subjects

Supplemental Table 2; Single regression analyses of dedifferentiation score with C-peptide index and diabetes duration in each group

Supplemental Table 3; The features of the subjects analyzed in Figure 4 and the classification into 3 comparison sets

Supplemental Table 4; Antibodies used for immunohistochemistry

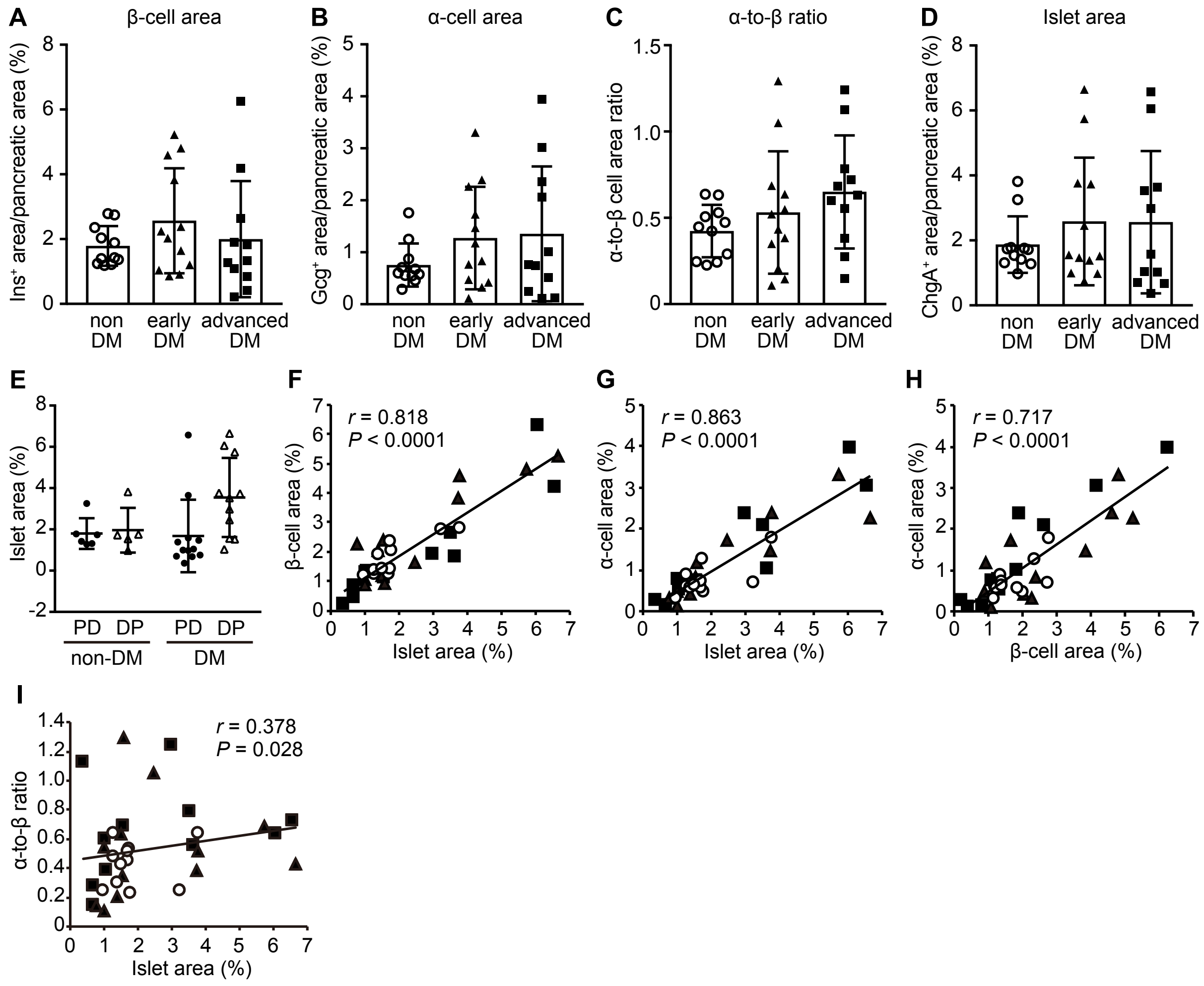

Supplemental Figure 1. Morphometric analysis of islets in pancreatic tissue sections from non-diabetic subjects and diabetic subjects

Comparisons of (A) β-cell area, (B) α-cell area, (C) ratio of α-cell area to β-cell area and (D) islet area, calculated as ChgA-immunoreactive area relative to total pancreatic section area. Data are means ± SD. n=11 for non-DM, n=12 for early-DM, n=11 for advanced-DM. We performed single regression analysis for comparisons between morphometric parameters (Spearman's r). (E) Comparison of islet area between distinct portions of the pancreas in each group. PD, pancreatoduodenectomy denoted by closed circles. DP, distal pancreatectomy denoted by open triangles. (F) Correlation between β-cell area and islet area. (G) Correlation between α-cell area and islet area. (H) Correlation between α-cell area and β-cell area. (I) Correlation between α-to-β cell area ratio and islet area. Open circles, non-DM control subjects. Closed triangles, early-DM subjects. Closed squares, advanced-DM subjects.

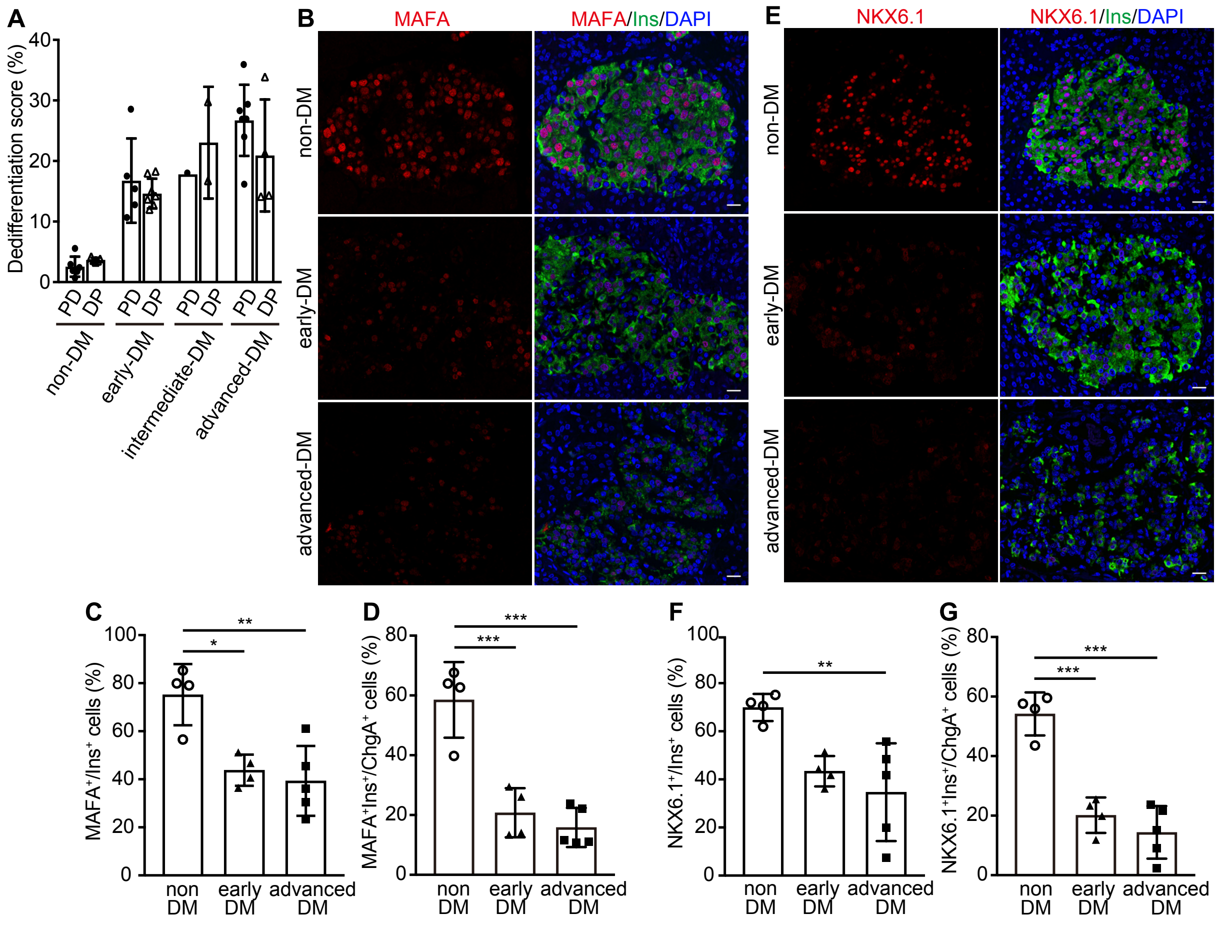

Supplemental Figure 2. The percentages of dedifferentiated cells in distinct pancreatic portions and analysis of two transcription factors, MAFA and NKX6.1

(A) Dedifferentiation scores in distinct portions of the pancreas in each group. PD, denoted by closed circles. DP, denoted by open triangles. (B) Immunofluorescence of pancreatic islets with MAFA (red), Insulin (green) and DAPI (blue). Scale bars, 20 μm. Quantitative analysis of MAFA and insulin double-positive cells normalized by (C) number of insulin-positive cells and (D) number of ChgA-positive cells in the pancreatic sections from subjects in each group. (E) Immunofluorescence with NKX6.1 (red), Insulin (green) and DAPI (blue). Scale bars, 20 μm. Quantitative analysis of NKX6.1 and insulin double-positive cells for (F) insulin-positive cells and (G) ChgA-positive cells in the pancreatic sections from subjects in each group. Data are means ± SD (n=4 for non-DM and early-DM, respectively. n=5 for advanced-DM). *, P<0.05; **, P<0.01; ***, P<0.001 by one-way ANOVA followed by Bonferroni's post hoc test.

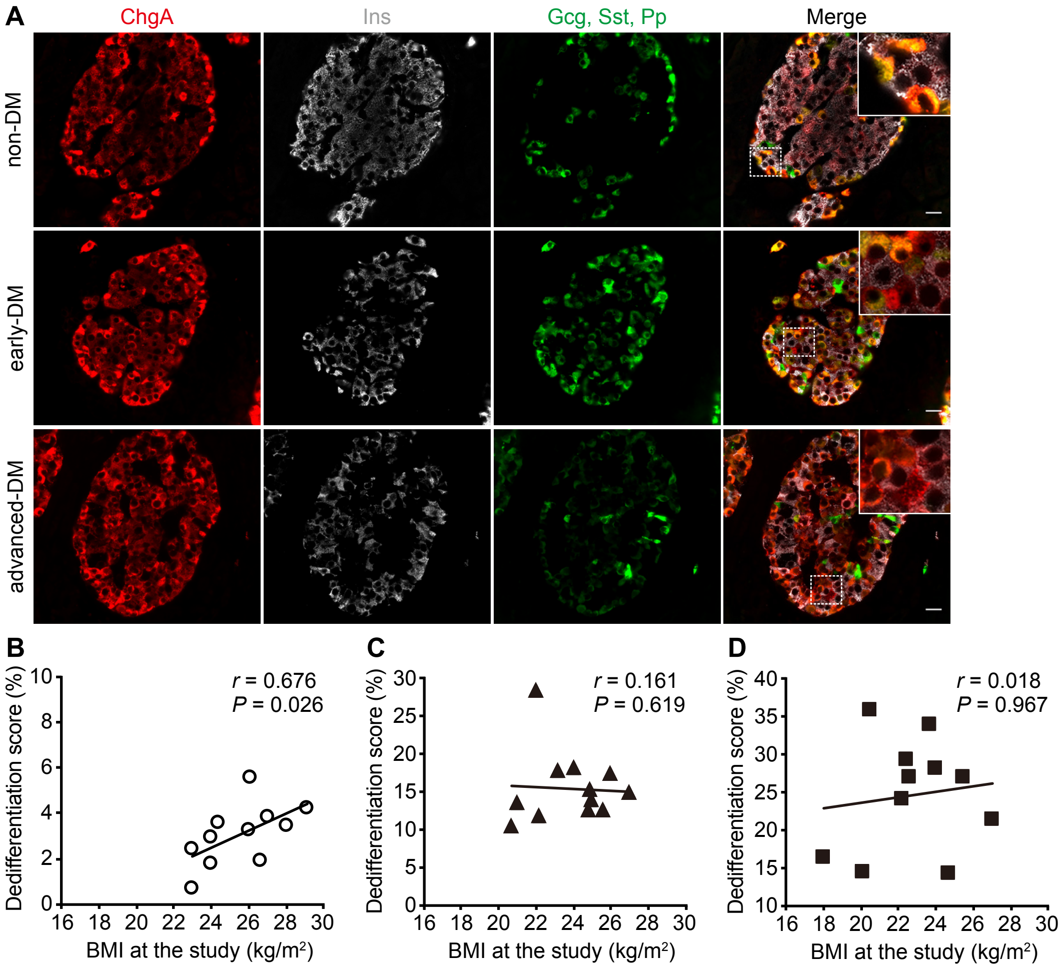

Supplemental Figure 3. Fluorescent image of islets separately stained for insulin and other pancreatic hormones separately in endocrine cells and correlations of dedifferentiation score with BMI for each group

(A) Representative images of pancreatic islets immunostained with ChgA (red), insulin (Ins, gray) and hormone cocktail (glucagon, somatostatin, pancreatic polypeptide) (green). Insets demonstrate representative cells. Scale bar, 20 μm. The immunohistochemical analysis was performed in 3 subjects of each group. Single regression analysis (Spearman's r) for correlation between BMI prior to pancreatectomy and dedifferentiation scores. The analysis was performed for each group, (B) non-DM (n=11), (C) early-DM (n=12) and (D) advanced-DM (n=11).

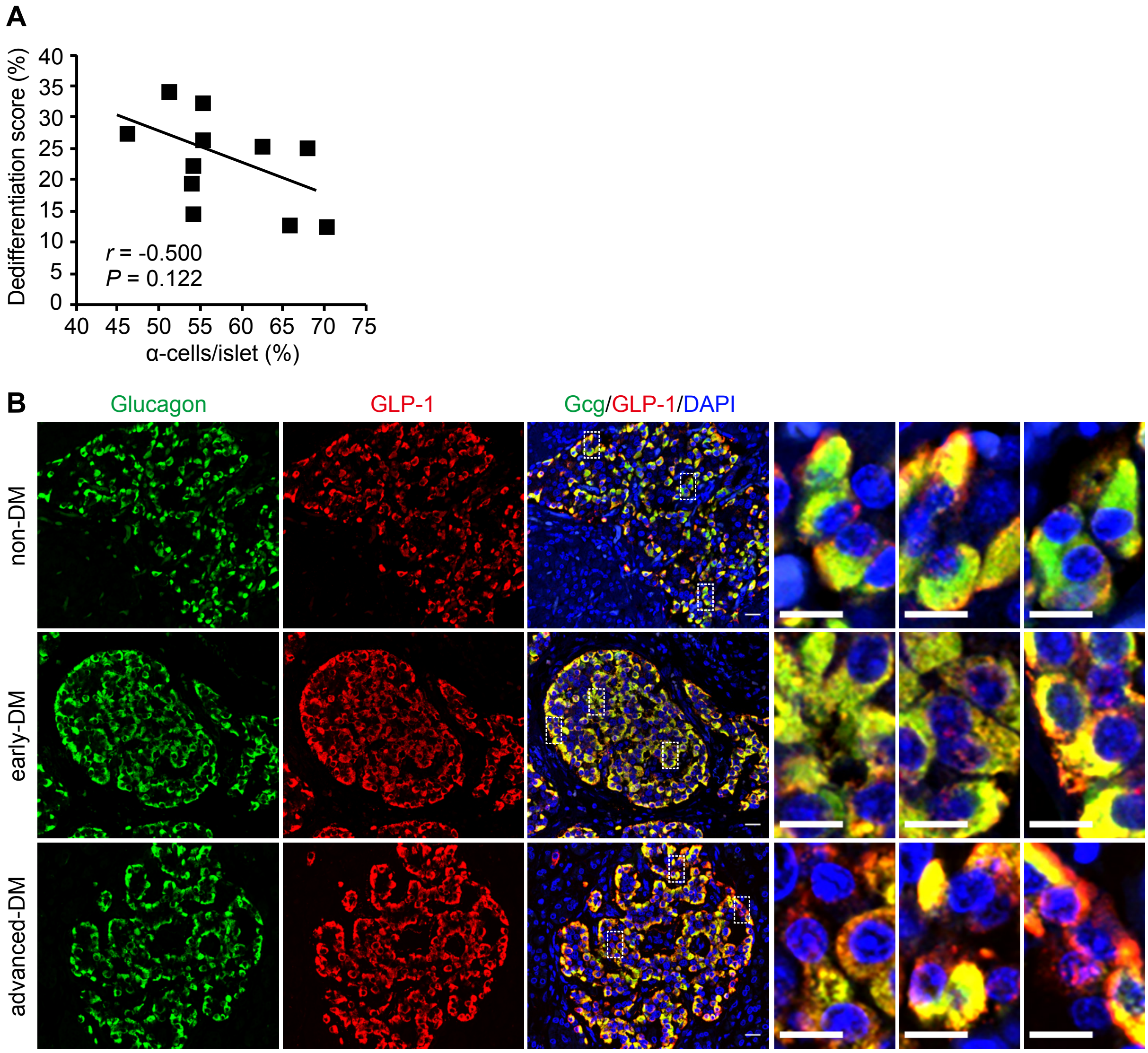

Supplemental Figure 4. Alterations in α-cells with disease progression

(A) Correlation between dedifferentiation score and α-cells/islet in advanced-DM subjects (n=11) (Spearman's r). (B) Representative immunofluorescence images of pancreatic islets with glucagon (green), GLP-1 (red) and DAPI (blue). Scale bars, 20 μm. The high magnification pictures show representative cells in the islets. Scale bars, 10 μm. The immunohistochemical analysis was performed in non-DM (n=5), early-DM (n=6) and advanced-DM (n=8).

Supplemental Table 1. Clinical characteristics of individual study subjects

CaseNo.	Gender, M/F	Age, years	Operative procedure, PD/DP	Clinical diagnosis	Diabetes duration, years	Maximum BMI, kg/m^2	BMI at the study, kg/m^2	HbA1c, % (mmol/mol)	FPG, mmol/l	F-CPR, nmol/l	CPI, nmol/mmol	Complication(s)	Diabetes Treatment
Non-DM													
1[a,b]	F	30	DP	NET	N/A	26	26	5.4 (36)	5.4	0.81	0.15	N/A	N/A
2[a,b]	M	62	PD	DPC	N/A	27	24	N/A*	5.2	N/A	N/A	N/A	N/A
3	M	58	PD	IPMN	N/A	28.5	26.6	6.1 (43)	5.4	N/A	N/A	N/A	N/A
4[a,b]	M	68	PD	CC	N/A	26	23	5.2 (33)	5.7	N/A	N/A	N/A	N/A
5[c]	M	77	PD	CC	N/A	27	23	5.2 (33)	5.2	N/A	N/A	N/A	N/A
6[a,c]	M	58	DP	PCY	N/A	25.4	24.4	5.6 (38)	5.3	N/A	N/A	N/A	N/A
7	M	73	PD	IPMN	N/A	27.8	26.1	5.9 (41)	4.8	N/A	N/A	N/A	N/A
8[b]	M	60	DP	PC	N/A	28	28	5.8 (40)	5.6	1.15	0.207	N/A	N/A
9[a]	M	66	PD	PC	N/A	25.3	24	5.9 (41)	5.8	0.61	0.105	N/A	N/A
10[a,c]	M	58	DP	NET	N/A	31.4	29.1	5.8 (40)	5.3	0.66	0.125	N/A	N/A
11	F	61	DP	NET	N/A	34.2	27	5.9 (41)	5	0.36	0.073	N/A	N/A
Early-DM													
12[b]	M	71	DP	PC	5	28	25	6.4 (46)	7	N/A	N/A	N/A	Diet
13[a,b]	M	70	DP	PC	3	26	21	6.8 (51)	6.8	N/A	N/A	N/A	Diet
14[a]	M	63	PD	DPC	3	26	26	7.6 (60)	5.3	N/A	N/A	N/A	Diet
15[a,c]	M	67	PD	CC	4	25.6	22	6.7 (50)	7	N/A	N/A	N/A	Diet
16[a,b,c]	M	66	DP	IPMN	3	29	27	6.3 (45)	5.8	N/A	N/A	N/A	Diet
17[a,b]	M	62	DP	PC	5	25	24	6.9 (52)	6.3	N/A	N/A	N/A	Diet
18	M	80	PD	CC	3	25.4	20.7	7.1 (54)	6.7	N/A	N/A	N/A	Diet
19	M	71	DP	PC	5	27	22.2	6.2 (44)	7.1	N/A	N/A	N/A	Diet
20[a,c]	M	62	DP	PCY	5	27.1	25.6	6.8 (51)	6.1	0.66	0.108	N/A	GI
21	F	76	PD	PC	4	25.2	24.9	7.3 (56)	6.8	0.53	0.078	N/A	DI
22	M	82	PD	CC	5	26.7	24.8	6.4 (46)	5.8	0.43	0.074	N/A	DI
23[a]	M	62	DP	NET	5	27	23.2	6.3 (45)	7.4	0.56	0.076	N/A	DI
Advanced-DM													
24[c]	M	44	PD	GC	16	28.2	18	7.6 (60)	9.5	0.36	0.038	RT	DI, SU
25[b]	M	81	DP	PC	15	30.5	27	7.5 (58)	6.9	0.7	0.101	RT	I
26	M	73	DP	PC	17	26	20.1	6.2 (44)	6.3	0.18	0.029	NU	SU
27[a,b]	M	63	PD	PC	20	26	22.2	7.7 (61)	9.6	0.5	0.052	NU, RT	DI, GI, M, SU
28[a]	M	64	DP	IPMN	15	30.4	23.66	7.1 (54)	9.6	0.4	0.042	NU, RT, NP	GI, M, SU
29[a,b]	M	60	PD	CC	10	29.7	25.5	7.1 (54)	8.9	0.89	0.101	NEU	I
30	F	80	PD	PC	30	26.2	20.5	8.5 (69)	8.7	N/A	N/A	NU, RT	DI, GI, SU
31[a,b]	M	69	PD	CC	17	27.4	24	6.4 (46)	6.9	N/A	N/A	NU, RT, NP	I
32[c]	M	78	PD	DPC	20	26	22.4	6.6 (49)	9.1	0.36	0.04	RET	DI, I, M, SU
33[a,b]	M	70	PD	IPMN	22	25.2	22.6	8.1 (65)	9.5	0.1	0.01	NU, RT, NP	I
34[c]	M	64	DP	IPMN	10	25	24.72	7.7 (61)	9.8	0.4	0.04	NP	DI, I
Intermediate-DM													
35	F	76	DP	PC	10	26.4	26.4	8.1 (65)	7.6	0.43	0.057	N/A	GI, P
36	M	43	DP	IPMN	7	28.6	27.5	7.4 (57)	7.6	0.53	0.07	N/A	DI, M, SU
37	M	62	PD	NET	9	31.8	25.5	6.3 (45)	5.2	0.36	0.07	NU, RT	GI, SU

*This case received 75g-OGTT prior to the operation but lacked a preoperative HbA1c measurement; [a]The subjects in whom insulin/glucagon-double positive cells were analyzed; [b]The subjects in whom transcription factors were analyzed; [c]The subjects in whom amylase were analyzed.

Abbreviations: BMI, body mass index, CC, cholangiocarcinoma; CPI, C-peptide index; DI, DPP-4 inhibitor; DP, distal pancreatectomy; DPC, duodenal papilla cancer; FPG, fasting plasma glucose; F-CPR, fasting C-peptide immunoreactivity; GC, gallbladder cancer; GI, α-glucosidase inhibitor; I, insulin; IPMN, intraductal papillary mucinous neoplasia; M, metformin; NU, neuropathy; NP, nephropathy; NET, neuroendocrine tumor; P, pioglitazone; PC, pancreatic cancer; PCY, pancreatic cyst; PD, pancreatoduodenectomy; RT, retinopathy; SU, sulfonylurea

Supplemental Table 2. Single regression analyses of dedifferentiation score with C-peptide index and diabetes duration in each group

Dedifferentiation score vs.		CPI	Diabetes duration
Non-DM	*r*	-0.6	N/A
	P	0.35	N/A
Early/intermediate-DM	*r*	-0.7928	0.3691
	P	0.0397	0.1755
Advanced-DM	*r*	0.2857	0.4129
	P	0.4529	0.2068

Summary of single regression analysis (Spearman's r) for correlation between dedifferentiation scores and c-peptide index (n=5 for non-DM, n=7 for early/intermediate-DM, n=9 for advanced-DM), and diabetes duration (n=11 for non-DM, n=15 for early/intermediate-DM, n=11 for advanced-DM). The analysis was performed for each group. CPI, C-peptide index.

Supplemental Table 3. The features of the subjects analyzed in Figure 4 and the classification into 3 comparison sets

	Case No.	Gender (M/F)	Age (years)	Operative procedure	Clinical diagnosis	Islet area (%)	Dedifferentiation score (%)
Set 1							
A. non-DM	5	M	77	PD	CC	3.3	0.7
B. early-DM	16	M	66	DP	IPMN	3.7	14.8
C. advanced-DM	32	M	78	PD	DPC	3.7	29.2
Set 2							
D. non-DM	6	M	66	DP	PCY	1.8	3.6
E. early-DM	20	M	62	DP	PCY	2.5	12.5
F. advanced-DM	34	M	64	DP	IPMN	3	14.2
Set 3							
G. non-DM	10	M	58	DP	NET	1.5	4.2
H. early-DM	15	M	67	PD	CC	1.4	28.3
I. advanced-DM	24	M	44	PD	GC	1.1	16.2

CC, cholangiocarcinoma; DP, distal pancreatectomy; DPC, duodenal papilla cancer; GC, gallbladder cancer; IPMN, intraductal papillary mucinous neoplasia; NET, neuroendocrine tumor; PCY, pancreatic cyst; PD, pancreatoduodenectomy

Supplemental Table 4. Antibodies used for immunohistochemistry

Primary antibodies				
Antigen	Species (Clone No.)	Source	RRID/Cat. No.	Dilution
Insulin	Guinea pig	Agilent, Santa Clara, CA, USA	AB_2800361/IR002	premixed
Glucagon	Mouse (79bB10)	Sigma-Aldrich, Saint Louis, MO, USA	AB_259852/G2654	1:2,000
Glucagon	Rabbit	CST Japan, Tokyo, Japan	AB_659831/#2760	1:100
Somatostatin	Rabbit	Abcam, Cambridge, UK	AB_11158517/ab108456	1:200
Pancreatic polypeptide	Rabbit	Phoenix Pharmaceuticals, Burlingame, CA, USA	NA/H-054-02	1:200
Chromogranin A	Goat	Santa Cruz Biotechnology, Dallas, TX, USA	AB_2276319/sc-1488	1:100
Chromogranin A	Mouse (LK2H10)	Merck Millipore, Burlington, MA, USA	AB_2081136/MAB5268	1:100
Amylase	Mouse (AMY-7)	Sigma-Aldrich	NA/SAB4200673	1:100
MAFA	Rabbit	Bethyl, Montgomery, TX, USA	AB_1279486/IHC-00352	1:200
NKX6.1	Rabbit	CST Japan	AB_2722625/#54551	1:1,000
GLP-1	Rabbit	Abcam	AB_447206/ab22625	1:1,000
Secondary antibodies				
Antigen	Species	Source	RRID/Cat. No.	Dilution
Guinea pig	Donkey (FITC-conjugated)	Jackson ImmunoResearch, West Grove, PA, USA	AB_2340453/706-095-148	1:200
Guinea pig	Donkey (Cy3-conjugated)	Jackson ImmunoResearch	AB_2340460/706-165-148	1:500
Guinea pig	Donkey (DyLight405-conjugated)	Jackson ImmunoResearch	AB_2340470/706-475-148	1:500
Mouse	Donkey (FITC-conjugated)	Jackson ImmunoResearch	AB_2340792/715-095-150	1:200
Mouse	Donkey (Cy3-conjugated)	Jackson ImmunoResearch	AB_2340813/715-165-150	1:500
Rabbit	Donkey (FITC-conjugated)	Jackson ImmunoResearch	AB_2315776/711-095-152	1:200
Rabbit	Donkey (Cy3-conjugated)	Jackson ImmunoResearch	AB_2307443/711-165-152	1:500
Goat	Donkey (Cy3-conjugated)	Jackson ImmunoResearch	AB_2340411/705-165-003	1:500

FITC, fluorescein isothiocyanate; Cy3, cyanine dye 3
